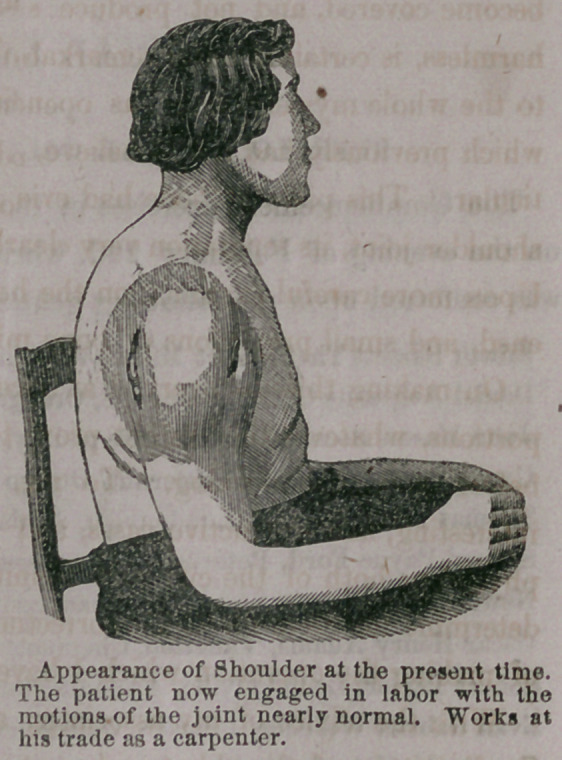# Clinical Remarks upon Surgical Cases in the Buffalo General Hospital—Exsection of the Head of the Humerus—Severe Injury—Erysipelas

**Published:** 1864-03

**Authors:** J. F. Miner


ART. V.—*Clinical Remarks upon Surgical Cases in the Buffalo General Hospital—Exsection of the Head of the Humerus—Severe Injury—Erysipelas.* By J. F. Miner, M. D.

Gentlemen:—I have the pleasure of presenting before you a case, which some of you have seen before. M. B. was the inmate of the Hospital of the Sisters of Charity about one year since, and your attention was then called to the remarkable condition of the shoulder joint by Prof. Moore. I have no knowledge of the views he entertained, or of the opinions of the numerous surgeons who have been consulted by this patient. The case first came under my observation something more than two years since—a single consulation at my office, and expression of desire to place himself under my care, being all that I knew of it, until after a great many efforts with private individuals and public institutions, he has come at last, without material change, and is willing to submit to whatever is proposed for his recovery. The shoulder joint has been the seat of the disease, and a great variety of opinions entertained as to the nature of the malady, almost every surgeon having a way of his own, of explaining the condition present. The shoulder has been immensely distended; the ligaments and tendons relaxed, and the head of the humerus easily dislocated from the glenoid cavity, moving in all directions with perfect ease. He has still had some use of the hand and arm, without ability to raise it, but able to use the forearm, while the arm hung by his side. It has never been painful or tender on pressure; has not disturbed the general health. It has never manifested tendency to suppurate, and even when opened it has soon healed, if allowed to do so. It has received treatment from several surgeons, but nothing has ever been accomplished by any, while some have given the disease a name, and declined treatment, or advised non-interference. I understand that the case has been looked upon generally, as entirely hopeless. It has been reserved for us to discover the true cause of the condition which you observe, and is no reflection upon the knowledge or ability of those who have preceded, when I intimate that they remained ignorant of the causes which have produced this condition of the shoulder joint.

When this patient was admitted to this Hospital he had exhausted all other resources; he was willing to suffer any operation which might be advised, and I was also ready to institute almost any course of treatment which would offer any hope of success. With the view of reducing the immense distension, and of creating inflammation and healthy suppuration, I at once made free openings, and three or four quarts of serum, and feebly organized fibrin, resembling whey, and curd, were removed from around the joint. The cavity containing this had before been opened, sometimes with an exploring trocar, sometimes with a lancet, always with the greatest caution; now it was opened by an incision four or five inches in length. And after removal of all foreign material the place was filled with charpie—pledgets of cotton cloth, with the view of inducing inflammation, and of keeping a free exit for serum or other accumulation. These openings were on either side of the articulation, and sufficiently free to admit the hand. By probing this opening with the hand we have discovered what could not have been known by any other process. The hand or the finger is the most improved probe, and though you cannot get the lead mark, or friction sound, you can yet sometimes obtain a knowledge of the condition of things, not to be gained by any other instrument. Yesterday in making some explorations in the cavity, a hard unyielding mass was found upon the wall of the posterior cavity, and after considerable force with the hand and a little dissection the bone which I here present, was found enclosed in fibrous tissue, encysted, as much so as any foreign substance could be. It is near an inch in length and rounded after the style of a large bean. How long it had been in this situation is not important, but that it should thus become covered, and not produce suppuration, but remain comparatively harmless, is certainly quite remarkable. It has been preserved as the key to the whole mystery, and has opened up to better understanding a case which previously had not, I believe, been understood in any essential particular. This piece of bone had evidently been attached to the bones of the shoulder joint, its separation very clearly indicated disease of the bony tissue. Upon more careful examination the head of the humerus was found roughened, and small projections of bone might be distinctly felt upon the neck.

On making this discovery, I at once proposed exsection of the diseased portions, whatever they might prove to be, and with this view he has come before you this morning. To me, this appears one of the most rare, interesting, and instructive cases, and I am glad to see present so many physicians both of the city and vicinity, who are invited to examine, and determine for themselves, the correctness of the explanation and the propriety of making the operation which I have proposed.

With the advice of my associates, and also of Dr. B. H. Colegrove, of Sardinia, one of the oldest and most experienced Surgeons in Western New York, who is with us this morning, I now propose to exsect the head of humerus and such other portions of bone as may be found diseased. I am going to disregard all surgical teaching in the manner of operation, and make it after my own style, a fashion applicable perhaps only to this particular case.

A flap is generally raised over the most prominent part of the shoulder, not unlike that made in amputation at the shoulder joint, and the joint fully exposed. Other plans of operation are also practiced. I have already a large opening posteriorly through which I now protrude the end of the bone, which is not bound by ligaments, tendons and muscles, as in conditions of health, but is easily dislodged from the glenoid cavity, and without great force projected through the opening, a little enlarged, which has served us so admirably for examination, and is now made to answer a double purpose. The operation has not proved difficult, and has been accomplished with nearly as great facility as by the more common method. The glenoid cavity appears to be healthy, and all unhealthy portions of the humerus have been removed including the head and anatomical neck; the remainder appears healthy, Inflammation under ordinary circumstances might be feared, but in this case there is little danger; indeed the tissues around this joint are incapable of taking on active inflammation; it has thus far resisted everything calculated to increase inflammatory action. We shall apply simple dressings; rest the fore arm in a sling, and report to you our success, when time shall have determined how much we have gained for our patient.

Outline of Shoulder before operation.

Appearance of Shoulder at the present time. The patient now engaged in labor with the motions of the joint nearly normal. Works at his trade as a carpenter.

*Erysipelas.*—There are two cases in the ward, to which I will ask your attention but one moment—two cases of Erysipelas. The one comes on after a severe injury caused by falling forty feet into a stone quarry; the other is idiopathic, and has gently caught hold of the patient's ear and nose. I thought you might some of you be interested to see the appearance—the outer manifestations of the disease. As we have not time for other considerations, you will desire to know at least what treatment we adopt. Nitrate of silver, sulphate of zinc, acetate of lead, sulphate of copper, persulphate and perchloride of iron, and a great many other medicines have been suggested and recommended as applications for Erysipelas; and it has been claimed that they prevent the spread, control the inflammation, relieve the pain, &c., &c. Muriated tincture of iron, externally and internally has been urged as all important. These are all fashionable with many practitioners, and probably they are productive of no great harm; I think they have little if any influence. Warm water dressings externally; opium, if necessary to allay pain and procure sleep; adequate support in cases of great depression, is all so far as I know and believe, which you can do for Erysipelas; and the result will be favorable almost invariably, except in some severe forms of epidemic disease.

## Figures and Tables

**Figure f1:**
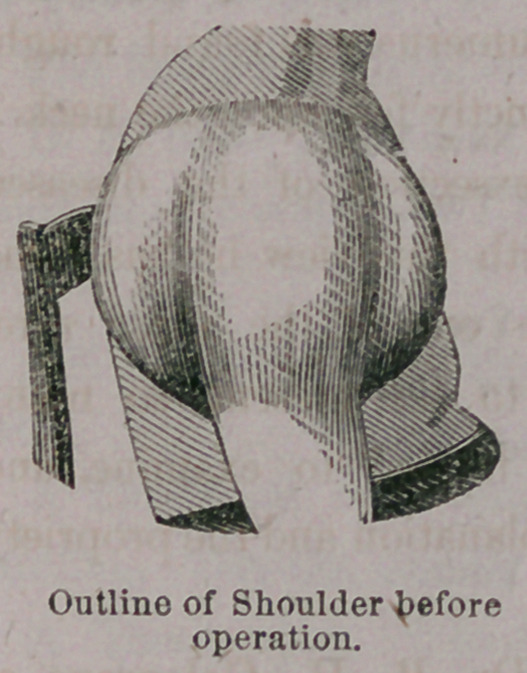


**Figure f2:**